# Desmoplastic small round cell tumor: an update of current management practices

**DOI:** 10.1186/s43046-025-00276-0

**Published:** 2025-04-21

**Authors:** Ahmed Gawash, Alexa Simonetti, Brandon J. Goodwin, Alissa Brotman O’Neill

**Affiliations:** 1 Department of Surgery, Rowan-Virtua School of Osteopathic Medicine, Stratford, NJ USA; 2 Lumina Institute, Cream Ridge, NJ USA; 3 Futures Forward Research Institute, Toms River, NJ USA

**Keywords:** Desmoplastic small cell round tumor, HIPEC, Surgery, Radiation, Chemotherapy

## Abstract

**Background:**

Desmoplastic small round cell tumor (DSRCT) poses a diagnostic challenge, initiating with imaging techniques like ultrasound, CT, MRI, and PET scans, with CT being the primary choice for abdominal tumor visualization. Despite advances, the absence of a standardized staging system complicates the diagnostic process.

**Methods:**

A comprehensive literature review and search strategy, encompassing databases like PubMed and Cochrane was performed.

**Results:**

Systemic chemotherapy, notably the P6 regimen, is the cornerstone of DSRCT treatment, while other options, including CDK4/6 inhibitors, antibody–drug conjugates, and anlotinib present varying efficacy. A novel chemotherapeutic agent, ONC-201, exhibits promise, inhibiting tumor growth in preclinical models. Surgical management, encompassing cytoreductive surgery and hyperthermic intraperitoneal chemotherapy (HIPEC), reveals improved survival rates, particularly when combined with chemotherapy. Whole abdomen radiotherapy (WART) emerges as a post-chemotherapy treatment option, despite potential complications. A study employing whole abdominopelvic intensity-modulated radiation therapy (WAP-IMRT) suggests reduced radiation toxicity compared to conventional WART. Immunotherapy, targeting receptors such as B7-H3, GD2, EGFR, HER2, tyrosine kinase inhibitors, and androgen receptors is a promising avenue, especially in younger populations, given its favorable long-term effects. Additionally, the inclusion of CCDN1 genomic alterations further informs potential targeted therapies for DSRCT.

**Discussion:**

This comprehensive review provides a current understanding of DSRCT diagnosis and treatment modalities, highlighting the ongoing challenges and promising avenues for future research. The integration of personalized approaches, novel chemotherapeutic agents, and evolving immunotherapy strategies holds the potential to enhance outcomes for individuals with DSRCT.

## Introduction

Desmoplastic small round cell tumor (DSRCT) is a rare and deadly malignancy [1]. This mesenchymal neoplasm primarily afflicts a distinct patient population, often affecting adolescents [2] and young adults who are male [2] with an age-adjusted incidence of 0.3 per million [3]. The neoplasia’s unique histopathological profile is hallmarked by the presence of small, round, undifferentiated cells embedded within an abundant desmoplastic stroma [4]. The prognosis of DSRCT patients is very poor, overall survival is approximately 30% to 55% despite chemotherapy, radiotherapy, and aggressive surgical resection [5, 6].

Genetically, DSRCT is characterized by a specific and recurring chromosomal translocation event. The hallmark genetic aberration in DSRCT involves the fusion of two distinct genes: the Ewing sarcoma gene (EWSR1) and the Wilms tumor gene (WT1), which is expressed as t(11;22)(p13;q12) [2, 7, 8]. This fusion gene is considered a defining molecular feature of DSRCT and plays a pivotal role in the pathogenesis of the disease [9]. The exact mechanism by which the EWSR1-WT1 fusion contributes to the development of DSRCT is not fully understood, but it is believed to function as an oncogenic transcription factor [9]. The zinc finger domain of the WT1 acts with affinity with certain promoter domains influencing the expression of various downstream proteins such as PDGFA, PAX2, IGF-1 receptor, and EGFR [9]. This is believed to be due to the zinc-finger domain of the WT1 which acts with certain promoter domains influencing the expression of various downstream proteins such as PDGFA, PAX2, insulin-like growth factor 1 receptor, epidermal growth factor receptor, IL2 receptor beta, BAIAP3, MLF1, TALLA-1, LRRC15, and ENT [9]. The EWSR1 portion of the fusion gene is known to interact with various cellular proteins involved in gene regulation and transcription [10]. The WT1 portion encodes for a transcription factor that has an essential role in the normal development of the urogenital system [11]. In DSRCT, the fusion of EWSR1 and WT1 likely leads to the dysregulation of important cellular processes, including gene expression and cellular growth control [12]. This genetic alteration is thought to be a key driver of the unique histological characteristics and aggressive behavior of DSRCT [13]. While the EWSR1-WT1 fusion event is a defining feature of DSRCT, it is worth noting that not all individuals with this fusion gene develop the disease, suggesting that additional genetic and environmental factors may contribute to disease susceptibility and progression [14]. Originating predominantly in the abdominal and pelvic cavities, DSRCT frequently infiltrates vital structures, including the peritoneum, omentum, and mesentery [15].

In the landscape of oncology, where individualized care and precision medicine are increasingly emphasized, DSRCT's rarity and the inherent difficulties in understanding its biology have led to it being overlooked or misdiagnosed [16]. The urgency of addressing this rare disease has catalyzed significant advances in recent years. Breakthroughs have been made in molecular research, elucidating its genetic underpinnings, specifically the identification of the EWSR1-WT1 fusion gene as a hallmark feature of DSRCT [12]. This discovery not only enables more accurate diagnosis but also opens avenues for targeted therapies [12]. Furthermore, the development of sensitive imaging modalities like positron emission tomography-computed tomography (PET-CT) and magnetic resonance imaging (MRI) has greatly improved early detection and staging [17]. Additionally, emerging therapeutic strategies, including hyperthermic intraperitoneal chemotherapy (HIPEC) [18], immunotherapies [19], and targeted therapies [20], are transforming the treatment landscape. These advances represent a paradigm shift in DSRCT management, offering renewed hope for more effective and less toxic treatment options, and are pivotal in enhancing patient outcomes and quality of life.

While DSRCT may be infrequent, its clinical course is anything but trivial. Its rapid growth, propensity for metastasis, and predilection for young individuals [21] necessitate immediate and comprehensive medical intervention. DSRCT has a very poor prognosis with a 5-year survival rate of less than 15%. Approximately 60–70% of patients with DSRCT die within 3 years of diagnosis [22, 23] and overall survival is approximately 30–55%, despite the use of aggressive multimodality treatments such as chemotherapy, radiotherapy, and aggressive surgical resection [5, 6, 23–25]. Recent years have witnessed a surge in clinical trials [20], innovative treatment strategies, and novel therapies [19] targeting molecular vulnerabilities specific to DSRCT. From targeted therapies to immunomodulatory approaches, and the promise of combination regimens, we aim to provide a comprehensive overview of the current state-of-the-art in DSRCT management.

A retrospective cohort study conducted in 2022 evaluated the incidence and survival rates of patients diagnosed with DSRCT in the USA. Data was obtained from the Surveillance, Epidemiology, and End Results (SEER) 9 Cancer Registry which reported 154 cases of DSRCT from 1975 to 2018, with 94% of the cases occurring after the year 2000. The study population was 79.8% males and 61% white, with the majority of patients being less than 60 years old [26]. Therefore, the clinical presentation of DSRCT occurs predominantly in males around 30 years old and consists of a large intra-abdominal, pelvic, or retroperitoneal mass with metastasis to the lungs, lymph nodes, and liver [27, 28]. Tumors outside of the abdomen are rare.

## Methods

This review was conducted to produce an updated list of treatment techniques for DSRCT. The review process and paper selection were conducted as follows.

Initial search strings consisted of “DSRCT AND Treatment” and “DSRCT AND management” to familiarize reviewers with the current and available literature. Once selecting the treatment methods (diagnosis, chemotherapy, surgery, radiation therapy, immunotherapy), individual article searches were conducted for each section. PubMed and Cochrane were the primary databases that were searched, with the majority of the articles being selected from PubMed, as Cochrane results were limited. Initially, a year range of 2020–2023 was applied in an attempt to gather the most current literature. There was not enough literature available in this time frame for any of the chosen treatment methods, therefore year ranges were expanded as needed to collect the most updated and relevant treatment methods.

The majority of the available literature consisted of case reports and retrospective database studies. Articles that detailed group treatments were chosen over individual case reports in an effort to standardize the success of the different management plans. Data was extracted from the selected studies which included information regarding study design, sample size, diagnostic methods, treatment modalities, outcomes, and key findings related to DSRCT. The findings from the selected studies were summarized and analyzed to provide a comprehensive overview of the current knowledge regarding DSRCT treatment and management modalities.

## Results

### Diagnosis

Diagnosis begins with imaging to visualize the presence of a tumor and is confirmed by biopsy. Imaging techniques include ultrasound, computed tomography (CT), magnetic resonance imagining (MRI), and positron emission tomography PET scan [28]. CT imaging has been shown to be the standard for initial tumor visualization of the abdominal area using intravenous contrast, whereas hepatic and pelvic lesions are better seen with MRI [29]. Imaging often underestimates disease severity due to the metastases developing 1–2 mm sheets of tumor [29]. Biopsy is conducted using fine needle aspiration (FNA) [30]. FNA uses a very thin needle and syringe to extract cells, tissue, or fluid from an area in the body and is especially useful for biopsying deep lesions. FNA aspirates of DSRCT show loose clusters of cells that can be uniform or slightly pleomorphic with high N:C ratios, as well as hypercellular small round or kidney-shaped cells with irregular hyperchromatic nuclei, molding, and poorly preserved cytoplasm [27]. FNA aspirates of DSRCT show loose clusters of cells that can be uniform or slightly pleomorphic with high N:C ratios, as well as hypercellular small round or kidney-shaped cells with irregular hyperchromatic nuclei, molding, and poorly preserved cytoplasm [27]. A characteristic feature of DSRCT tumor cells is small nests of tumor cells with desmoplastic stroma, which can be seen on an MGG stain [27]. Less common, but still relevant findings include acinar-like structures, pseudorosettes, and myxoid matrices in smears [27]. DSRCT exhibits an unknown histogenesis [27]. The diagnostic process lacks a universally standardized staging system designed specifically for DSRCT [29]. As of 2016, Hayes-Jordan et al. created a new staging system that was being used on a trial basis, but no further updates have been provided on the validity of the new staging system [29].

### Chemotherapy

Systemic chemotherapy is the initial treatment upon DSRCT diagnosis, prescribed in conjunction with other treatment modalities such as surgical debulking or radiation therapy [31]. The most common first-line chemotherapy option is the P6 regimen; a mixture of vincristine, doxorubicin, cyclophosphamide, ifosfamide, and etoposide [31]. Second-line chemotherapy options include high-dose ifosfamide, temozolomide/irinotecan, and cyclophosphamide/topotecan combinations [32]. Tumor responsiveness to chemotherapy indicates whether or not the patient is a solid candidate for surgery [33].

A 2021 study by Jeong et al. evaluated the effectiveness of multiline chemotherapy treatments for DSRCT. The first line chemotherapy regimen used in all 14 patients was the P6 regimen, which demonstrated a disease control rate of 100%, an overall response rate of 57.1%, and a median time-to-progression of 9.9 months (95% CI, 7.2–11.7) [31]. It was discovered during follow-up sessions that 13 patients (92.9%) sustained disease progression despite the initial response, while the remaining patients received surgery after the delivery of the first line of chemotherapy. Out of the 13 patients whose disease progressed after first-line treatment, 10 received a second line of chemotherapy. Second-line chemotherapy treatments were cytotoxic and included ICE (ifosfamide, carboplatin, etoposide) and VIP (etoposide, ifosfamide, cisplatin). Second-line treatments demonstrated a disease control rate of 88.9%, an overall response rate of 0%, and a median time-to-progression of 3.5 months (95% CI, 1.4–8.6). Eight patients received a cytotoxic regimen as a third-line chemotherapy treatment, where the disease control rate was 75%, the overall response rate was 12.5%, and the median time-to-progression was 2.5 months (95% CI, 0.9–4.8). Results showed patients with a longer (greater than the median) time-to-progression after first-line treatment had a longer time-to-progression after second-line treatment (*P* = 0.72), and a longer time-to-progression after third-line treatment (*P* = 0.23), but these results were not statistically significant [31]. It was noted that the time to disease progression decreased after administering a subsequent line of chemotherapy.

A new chemotherapeutic agent being tested for its efficacy against DSRCT is ONC-201. The mechanism of action of ONC-201 is to isolate the death receptors (DR4 and DR5) to activate the TNF-related apoptosis-inducing ligand (TRAIL) pathway and induce apoptosis in cancer cells [34]. This method yielded results in bone tumors, therefore Hayes-Jordan et al. hypothesized that ONC-201 could be employed to inhibit tumor growth in sarcomas. Through using in vitro proliferation assays and in vivo orthotopic xenograft animal models of DSRCT, results showed that ONC-201 inhibited cell proliferation associated with caspase activation and tumor growth, suggesting it could be used clinically as a therapeutic agent to control DSRCT proliferation [34].

In 2022, Anderson et al. conducted a Phase II trial testing the efficacy of ONC-201 against neuroendocrine tumors, including DSRCT, in human populations. The treatment protocol for those with DSRCT consisted of ONC-201 administration at a dose of 625 mg orally once per week. DSRCT patients had rapid disease progression, with only 3 of 10 patients receiving ONC-201 therapy for over 6 months- one being over 12 months and the other being over 4 years. Results showed that ONC-201 produced mild and infrequent side effects. It was concluded that oral ONC-201 displayed some clinical benefit against DSRCT, and may be utilized best when used as an adjuvant after cytoreduction surgery, chemotherapy, and radiotherapy. The usage of ONC-201 requires more research and testing to prove if it would be a useful treatment option [35].

### Surgery

Surgical management of DSRCT is attempted through cytoreductive surgery, also known as debulking surgery [36]. The goal of debulking surgery is to remove the highest percentage of the tumor possible since the remaining cells can metastasize [37]. In a 2018 study by Subbiah*,* et al., 57% of the 187-person study population underwent a complete cytoreductive surgery [32]. These participants displayed a better overall survival rate (*P* = 0.006) than those who could not receive the surgery [32]. Results showed chemotherapy (log-rank *P* = 0.004) and complete cytoreductive surgery (log-rank *P* < 0.0001) demonstrated statistical significance and were associated with improved survival both on their own, and when partnered with other treatment modalities [32].

HIPEC is an additional surgical treatment option that is often performed immediately after cytoreductive surgery. The effectiveness of administering both techniques together has not yet been determined due to conflicting literature. The rationale behind performing HIPEC after cytoreductive surgery is to eliminate any micrometastases and free tumor cells that were not removed during surgery [37]. To administer HIPEC, drains are inserted in the abdominal cavity to allow for the perfusion of heated high-dose chemotherapy, which is then circulated for 30 to 90 min. The two methods for HIPEC include the open and closed abdomen technique, which pertains to the status of the laparotomy [37]. The range of tissue penetration depends on many factors including the drug, temperature, and time of intraperitoneal chemotherapy. At a dosage of 100 mg/m^2^ at 41 °C for 90 min, Cisplatin is the most common drug used for HIPEC [38].

In a retrospective study between 1991 and 2018, 100 patients were identified and their treatment method efficacy was analyzed [39]. HIPEC was administered to 15 patients during surgery. Researchers found no benefits of adding HIPEC as a treatment method, given the high percentage (88%) of the first disease recurrence site being the peritoneum (88%). This demonstrates that HIPEC is unable to eradicate microscopic residual disease. HIPEC did not show statistically improved survival rates (HR = 1.35, *p* = 0.65) [39]. HIPEC also lacked statistical significance in a similar 2018 study by Subbuah et al., although it is important to note researchers stated the study was not geared to properly measure the efficacy of HIPEC, as the study’s focus was on chemotherapy and complete cytoreductive surgery [32].

### Radiation therapy

Whole abdomen radiotherapy (WART) is another method used to treat DSRCT and is usually administered after chemotherapy, surgical debunking, and HIPEC [40]. One of the complications of WART is severe gastrointestinal and liver toxicity is common [40].

In a 2016 study by Pinnix, et al., medical records of patients with DSRCT who were treated with whole abdominopelvic intensity-modulated radiation therapy (WAP-IMRT) at MD Anderson from 2006 to 2010 were reviewed. Eight male patients underwent WAP-IMRT after receiving chemotherapy, and surgical debulking, and seven of them also had HIPEC. WAP-IMRT was administered once daily at a dosage of 30 Gy, in 20 1.5-Gy fractions with 6-MV photons, with four patients receiving an additional boost of 6–10 Gy to areas with residual disease [41]. Beams utilized per patient varied between 9 and 14, with all plans having one isocenter except for two, which required two isocenters- an upper and a lower. Treatment duration ranged from 25 to 31 days. Of the eight patients treated with WAP-IMRT, five experienced intra-abdominal recurrence in the treatment field within a mean time of 262 days, while two patients sustained disease recurrence outside the field, and the last is alive with no signs of disease. Final patient follow-ups were conducted over a range of 12–64 months; five patients were alive (four with active disease and one without disease), and three died of disease progression [41]. Of the eight patients, treatment failure of the abdomen occurred in five patients (68%), whereas treatment failure outside of the abdominal region occurred in three patients (37.5%), and isolated failure involving the mediastinum or thoracic vertebral body (T3) was present in two patients (25%). The one patient who remained alive with no evidence of recurrent disease completed radiation therapy 20 months prior to the last follow-up [41]. WAP-IMRT delivered 25% less doses of radiation to pelvic bones and lumbosacral vertebral bodies than standard radiotherapy treatment, suggesting it may be a better alternative compared to conventional WART due to the reduced radiation toxicity [41].

### Immunotherapy

An important factor for determining immunotherapy targets requires the target to be actively expressed on tumor cells and restricted in normal cell tissues. This is an important quality because it prevents the targeting of nontumor cells. Multiple DSRCT cell surface receptors have been identified as targets for immunotherapy. One of the most prevalent targets is B7-H3, which is the target of the monoclonal antibody 8H9 and is regulated by microRNA-29 [40]. Other identified but less prevalent targets include GD2, EGFR, and HER2. There are numerous clinical trials being conducted testing different immunotherapy treatments on these targets [40]. Using immunotherapy to treat DSRCT in younger populations would be advantageous due to its lack of negative long-term effects, especially when compared to other treatment modalities.

Compartmental delivery is a new development within immunotherapy. Delivering the therapeutic agent directly into the location of the tumor helps restrict systemic exposure and decrease toxicities caused by on-target-off tumor responses that are often seen with IgG radioimmunoconjugates [41]. In the case of DSRCT, intraperitoneal delivery would be the best compartmental delivery location in order to target the surface receptors of the tumor cells.

### Targeted therapy

Recent studies have also shown genomic alterations beyond the known EWSR1 rearrangement. Specifically, Gaidzik et al. found CCND1 genomic gain in two of their patients, and Cyclin D1 expression was detected in all seven tumors analyzed. Furthermore, the use of CDK4/6 inhibitors such as palbociclib has been explored, although clinical response remains modest, with a median progression-free survival of 4.5 months [42]. Furthermore, Boulay et al. found that inhibition of CCND1 by palbociclib resulted in a significant reduction of tumor burden in DSRCT patient-derived xenografts in vivo [43].

Another therapeutic strategy for DSRCT was discussed in Magrath et al. which demonstrated that palbociclib effectively reduced tumor growth in DSRCT xenograft models further supporting the potential of CDK4/6 inhibitors as a promising therapy [44].

HER2 antibody–drug conjugates, such as trastuzumab deruxtecan and trastuzamab emtansine, have also demonstrated efficacy against HER2-positive DSRCT. Zhang et al. revealed that in HER2-positive DSRCT, HER2-targeted antibody–drug conjugates resulted in substantial tumor regression in patient-derived xenografts, cell lines, and organoid models [45].

Magrath et al. also addressed the male predominance in DSRCT, noting that 80–90% of patients are males. Their hypothesis was that the androgen receptor (AR) may play a role in driving tumor growth, due to elevated testosterone levels in males. Their study demonstrated that AR is highly expressed in DSRCT compared to other fusion-driven sarcomas. They found that AR antagonists such as enzalutamide and flutamide reduced DSRCT growth. However, DSRCT cell lines were also found to form xenografts in female mice at the same rate as in males. Moreover, AR depletion did not significantly alter DSRCT growth in vitro. This all suggests that AR-targeted therapy may provide therapeutic benefit through an AR-independent mechanism, and AR independence may not explain the male predominance in DSRCT.47.

### Tyrosine-kinase inhibitor

In a case series by Jing et al., anlotinib, a multitarget tyrosine-kinase inhibitor showed promising results when combined with chemotherapy for the treatment of pediatric DSRCT. This therapy significantly reduced tumor size and improved progression-free survival in two patients, although one patient succumbed to post-operative complications. This drug is only available in China, but this case series introduces a potentially effective treatment option [46].

## Discussion

### Diagnosis

It is crucial to acknowledge the limitations of imaging modalities such as ultrasound, CT, and MRI in the detection of DSRCT, particularly in estimating disease severity due to the nature of metastases as small sheets of tumor [29]. This highlights the necessity of combining clinical and radiological findings with precise biopsy techniques for a definitive diagnosis.

Fine needle aspiration (FNA) is the preferred method for obtaining tissue samples for diagnosis [30]. Its utility is especially pronounced when dealing with deep-seated lesions. Therefore, clinicians should be aware of the critical role that biopsy plays in the accurate diagnosis of DSRCT and its potential implications for treatment decisions.

### Chemotherapy

Systemic chemotherapy remains the cornerstone of DSRCT management. The most commonly used first-line regimen, the P6 protocol, combines multiple agents and has shown promising results [31]. It is critical to underscore that the responsiveness of the tumor to chemotherapy is a significant determinant of a patient’s eligibility for surgery [33]. This emphasizes the importance of a multidisciplinary approach to treatment, where medical oncologists work closely with surgeons to tailor the therapeutic strategy to each individual’s condition.

The discussion also introduces ONC-201 as a novel chemotherapeutic agent being explored for its efficacy in DSRCT [34]. The results of preclinical studies and a Phase II trial highlight its potential to inhibit DSRCT proliferation [34]. While further research is needed to establish its role in clinical practice [35], such developments represent a shift towards more targeted and potentially less toxic treatment options. Furthermore, CDK4/6 inhibitors have recently shown promising options for targeting the molecular drivers of DSRCT [42, 44].

### Surgery

Surgery plays a vital role in DSRCT management, with cytoreductive surgery aiming to remove as much of the tumor as possible [36]. Studies indicate that complete cytoreductive surgery can improve overall survival rates [32]. The potential benefits of hyperthermic intraperitoneal chemotherapy (HIPEC) are introduced, but the effectiveness of combining it with cytoreductive surgery is an area of ongoing investigation. This underscores the need for further research to clarify the best practices in surgical management.

### Radiation therapy

Whole abdomen radiotherapy (WART) is discussed as an adjuvant treatment modality, typically administered after chemotherapy, surgical debulking, and HIPEC [40]. The dosage and timing of radiation therapy are crucial considerations, and further research is needed to optimize the use of this technique in DSRCT management.

### Immunotherapy

Immunotherapy represents an exciting frontier in cancer treatment, and potential targets for DSRCT have been identified. B7-H3, GD2, EGFR, and HER2 are among the targets being explored, and their specificity to tumor cells offers promise for immunotherapeutic interventions [40, 47]. This approach may hold particular advantages, especially for younger patients, with fewer long-term side effects compared to traditional therapies.

### Targeted therapy

Recent research highlights the potential of targeting AR, particularly given the elevated testosterone levels in males [48]. Also, HER2-targeted therapies are being explored with promising results in HER2-positive DSRCT [45]. Additionally, anlotinib, has shown encouraging results in early trials [46].

### Strengths and limitations

This study boasts several strengths, notably its comprehensive exploration of current desmoplastic small round cell tumor (DSRCT) management practices. The inclusion of a retrospective cohort study from the Surveillance, Epidemiology, and End Results (SEER) 9 Cancer Registry spanning several decades provides a valuable, extensive dataset that enhances our understanding of DSRCT incidence and survival rates. However, it is important to acknowledge the limitations inherent in the study. The retrospective nature of the cohort analysis introduces potential biases and issues related to data accuracy. Additionally, the rarity of DSRCT hinders the availability of large, randomized clinical trials, limiting the robustness of evidence on treatment efficacy. The evaluation of new therapies, like ONC-201, is primarily based on preclinical and early-phase clinical trials, warranting further extensive research to solidify their role in the clinical setting.

### Future directions

The promising results and ongoing research in DSRCT management provide clear guidance for future directions in the field. These include a deeper exploration of targeted therapies, such as ONC-201, to unravel their efficacy, mechanisms of action, and potential synergies with existing treatment modalities. Multimodal treatment strategies that optimize the combination of chemotherapy, surgery, radiation therapy, and immunotherapy are essential for improving overall survival and quality of life for DSRCT patients. Precision medicine needs to play a more prominent role, focusing on the genetic and molecular factors influencing DSRCT progression and treatment response to enable personalized therapeutic approaches. Enhancements in early detection and staging through advanced imaging and diagnostic techniques will further advance outcomes. Encouraging participation in clinical trials is vital to assess the safety and efficacy of novel therapies and ensure that the latest advancements benefit patients with DSRCT. Lastly, the study of long-term treatment effects, particularly in younger patients, is pivotal to optimizing therapeutic strategies and minimizing potential late side effects.

### Clinical implications

The findings and ongoing developments in DSRCT management carry significant clinical implications. Advanced diagnostic methods, which combine precise biopsy techniques with sophisticated imaging, ensure more accurate and timely DSRCT diagnoses, thus facilitating early interventions. The complex nature of DSRCT necessitates a multidisciplinary approach, involving medical oncologists, surgeons, radiologists, and pathologists to tailor treatment plans and optimize patient care. The emerging field of precision medicine allows for the customization of therapies based on the unique genetic and molecular characteristics of each patient’s DSRCT, potentially increasing treatment effectiveness. Integrating new chemotherapeutic agents and multimodal treatments, along with the optimization of surgery and radiation therapy, offers improved prospects for survival and long-term quality of life for DSRCT patients. Ongoing research and clinical trials provide hope for individuals affected by DSRCT, emphasizing the potential for breakthroughs in treatment strategies that may transform the management of this rare and aggressive malignancy. In summary, the clinical implications highlight the importance of staying informed about the latest developments in DSRCT management and ensuring that patients receive the most current and effective care tailored to their specific conditions.

## Conclusion

Desmoplastic small round cell tumor (DSRCT) presents a formidable challenge within the landscape of oncology, with its rarity and complex clinical characteristics. However, the current state of DSRCT management represents a paradigm shift in the approach to this malignancy. Our exploration of diagnostic methods, chemotherapy, surgery, radiation therapy, and immunotherapy reveals a multifaceted strategy that is reshaping the way we perceive and treat this disease. The development of targeted therapies and precision medicine holds the potential to improve overall survival and enhance the quality of life for individuals affected by DSRCT. While DSRCT remains a rare and formidable entity in oncology, the current progress and ongoing research underscore the importance of maintaining a dynamic and informed approach to patient care. With advancements in diagnosis and a shifting treatment landscape, we find ourselves on the cusp of transformative change in the management of this complex malignancy.

## Data Availability

There is no relevant data for this study.

## Abbreviations

EWSR1: Ewing sarcoma gene
WT1: Wilms tumor gene
DSRCT: Desmoplastic small round cell tumor
CT: Computed tomography
MRI: Magnetic resonance imaging
PET: Positron emission tomography
P6 regimen: Chemotherapy regimen combining vincristine, doxorubicin, cyclophosphamide, ifosfamide, and etoposide
ONC-201: Novel chemotherapeutic agent
HIPEC: Hyperthermic intraperitoneal chemotherapy
WART: Whole abdomen radiotherapy
WAP-IMRT: Whole abdominopelvic intensity-modulated radiation therapy
SEER: Surveillance, Epidemiology, and End Results
FNA: Fine needle aspiration
DR4: Death receptor 4
DR5: Death receptor 5
TRAIL: TNF-related apoptosis-inducing ligand
PDGFA: Platelet-derived growth factor A
EGFR: Epidermal growth factor receptor
HER2: Human epidermal growth factor receptor 2
ICE: Ifosfamide, carboplatin, etoposide
VIP: Etoposide, ifosfamide, cisplatin
